# Maternal Diet Influences Human Milk Protein Concentration and Adipose Tissue Marker

**DOI:** 10.3390/nu15020433

**Published:** 2023-01-14

**Authors:** Christoph Binder, Sabina Baumgartner-Parzer, Liliana-Imi Gard, Angelika Berger, Alexandra Thajer

**Affiliations:** 1 Comprehensive Center for Pediatrics, Department of Pediatrics and Adolescent Medicine, Medical University of Vienna, 1090 Vienna, Austria; 2 Division of Endocrinology and Metabolism, Department of Internal Medicine III, Medical University of Vienna, 1090 Vienna, Austria

**Keywords:** human milk, maternal diet, maternal body mass index, macronutrients, adiponectin, leptin, nutrition, preterm, individualized protein target fortification

## Abstract

(1) Background: Adequate protein intake plays an essential role in growth and neurodevelopment, especially in preterm infants. We investigated the effects of maternal diet and body mass index (BMI) on human milk (HM) composition. (2) Methods: HM samples were obtained from 136 lactating mothers (BMI: 18.0–36.7 kg/m^2^), of which 93% gave birth to preterm infants. Macronutrient content in HM was measured by mid-infrared transmission spectroscopy. Leptin and adiponectin were analyzed using appropriate ELISAs. Maternal diet was determined by 24-h recall. (3) Results: Significant positive associations were found between protein, fat, carbohydrate and energy intake, and levels of corresponding macronutrients in HM, especially in protein concentrations (*p* < 0.001). An increased protein intake was positively correlated with adiponectin (*p* < 0.001) and leptin (*p* = 0.035) in HM. Maternal BMI was positively associated with a higher protein level in HM (*p* < 0.05), as well as with a higher dietary protein intake (*p* < 0.05). (4) Conclusions: Knowledge of maternal diet and BMI impacting HM composition is essential to optimize the feeding of newborn infants. This is especially relevant in the nutritional management of preterm infants; it can be utilized in approaches to improve growth rates and the appropriate development of infants and to prevent obesity.

## 1. Introduction

Human milk (HM) is a highly complex fluid that represents the gold standard of nutrition for newborn infants and supports normal growth and development [1,2,3,4]. A HM diet is crucial for normal brain development, especially in preterm infants [5]. Previous studies showed that protein intake is positively correlated with neurodevelopment [6].

The composition of HM of each individual mother is unique and is influenced by many factors, including the mode of delivery; the gestational age (GA), birth weight and sex of the infant; the geographical or genetic background of the lactating mother; maternal health; maternal behavioral habits (e.g., smoking, alcohol); maternal age; maternal body mass index (BMI); stage of lactation; volume and frequency of breastfeeding, etc. [7,8,9,10,11,12,13,14,15]. Maternal diet has been reported to affect the HM composition, with conflicting results [10,16,17,18,19]. Furthermore, the effect of maternal diet on the HM composition of mothers of preterm infants has yet to be explored. A closer examination of the literature shows that single nutrients (e.g., fatty acids, vitamin C, vitamin E, retinol, calcium, magnesium, iron, zinc and copper) have been investigated in detail in the maternal diet, as well as in the HM profile [19,20,21,22,23]. Data on maternal diet, especially on maternal macronutrient dietary intake and the effect on the macronutrient HM composition, are missing [10].

Breastfeeding has multifactorial beneficial effects on the mother as well as on the infant, and it decreases the risk of developing obesity in early childhood [24,25,26,27,28]. However, the effect of maternal obesity on HM composition and the exact mechanism of obesity prevention through breastfeeding are unknown. Additionally, it is unclear whether an underweight mother has a different HM content compared to normal-weight lactating mothers, or overweight or even obese mothers. In summation, the literature exploring maternal weight status and the potential impact on HM composition is inconsistent [29,30,31,32]. In terms of HM and obesity prevention, the knowledge of the HM macronutrient composition, specifically protein concentrations, is essential; however, the levels of the regulatory adipose tissue hormones leptin and adiponectin are also crucial [33,34]. Thus, we explored the effect of maternal diet and maternal body weight on HM composition and adipose tissue hormones.

We hypothesized that the dietary intake of the lactating mother is reflected in the composition of expressed HM, with a particular emphasis on protein, which is an essential macronutrient for normal growth and neurodevelopment in infants. We also investigated the impact of maternal BMI on the HM composition and on the adipose tissue markers adiponectin and leptin.

## 2. Materials and Methods

### 2.1. Population

Between March 2016 and July 2022, a total of 136 lactating mothers and their preterm and term infants were recruited during their hospital stay at the General Hospital of Vienna, Medical University of Vienna. The study fulfilled the good practices of the Declaration of Helsinki and was approved by the Institutional Review Board of the Medical University of Vienna (EC: 2022/2015) on 5 February 2016. Informed consent was obtained from all participating mothers.

### 2.2. Human Milk Collection and Analysis

This study is a secondary analysis of a trial evaluating different types of HM processing and the impact on HM composition (EC: 2022/2015, [35]). We gathered 150 mL HM from each mother; all HM samples were obtained from mothers with sufficient lactation. The HM samples were collected through manual expression at the end of a single session of breastfeeding or through breast pumping into a sterile feeding bottle. All human milk samples from individual mothers were collected over 24 h and were pooled to limit HM variations caused by single breastfeeding sessions or circadian variability (day vs. night expressions). The HM samples were picked up directly after collection from the refrigerator in the ward. Mothers discharged from hospital were instructed to store and pool their HM samples in the refrigerator at home and to bring the samples to the Department of Pediatrics and Adolescent Medicine at the Medical University of Vienna the next day. At the maternity ward, the data on lactation days were collected and lactation day 1 started 24 h after birth. The lactation period was defined as days between birth and HM sample collection for each mother.

The macronutrients protein (true protein and crude protein), fat and carbohydrate, as well as energy and total solids, were determined using the Miris Human Milk Analyzer^TM^ (Miris AB, Uppsala, Sweden). This method uses mid-infrared transmission spectroscopy and was calibrated against standard methods [36,37,38,39,40,41]. HM analysis was performed according to the manufacturers’ recommendations [42]. The homogenization was performed using a Miris Ultrasonic Processor^TM^ (Miris AB, Uppsala, Sweden), and all samples were maintained at 40 °C with the Miris Heater^TM^ (Miris AB, Uppsala, Sweden) prior to measurement. A daily quality control, as well as calibration, checks and cleaning steps, were performed using a Miris Calibration Control Kit^TM^, Miris Check^TM^ and Miris Cleaner^TM^ (Miris AB, Uppsala, Sweden), according to the manufacturers’ instructions, prior to the analysis of the HM samples [42]. A sample volume of 3–6 mL was required to conduct duplicate measurements.

Adiponectin and leptin were analyzed using a leptin and a high-sensitivity adiponectin ELISA (enzyme-linked immunosorbent assays), both CE-certified and purchased from BioVendor (Brno, Czech Republic). The standard curve was prepared to cover a lower concentration range, and the incubation time of the samples with the antibody and conjugate was extended according to the manufacturer’s recommendations. The protocol used in the present study has previously been proven for leptin and adiponectin measurements in HM [43]. As recommended by the manufacturer, milk samples were centrifuged at 2500× *g* for 20 min at 4 °C to obtain skimmed milk prior to ELISA analysis. All samples were analyzed in duplicate.

### 2.3. Assessment of Maternal Diet

The nutritional evaluation of the maternal diet was determined with a 24-h recall. Throughout the interviews, mothers were asked about food and drinks consumed the previous day (within the last 24 h) prior to the HM collection. All nutritional interviews were performed by the same trained nutritionist to prevent any bias. At first, all 24-h recalls were completed on paper and were then translated into the NutriSurvey database (EBISpro, Stuttgart, Germany) by the trained nutritionist. The maternal micronutrients, macronutrients and total energy intake were estimated using the German Nutrient Database [44]. Daily nutrient and energy intake were analyzed, with particular focus on protein, fat and carbohydrates.

### 2.4. Statistical Analysis

All data were tested for normality and described as mean and standard deviation (SD), or median and interquartile range (IQR). The categorical variables were expressed as absolute frequencies or percentages. Comparisons were performed using non-parametric tests to ensure higher robustness. The Kruskal–Wallis analysis was followed by a Mann–Whitney U Test to compare continuous variables between the maternal BMI groups.

Correlations between maternal diet and HM composition were estimated with Spearman’s signed rank correlation coefficient (Spearman’s rank). A comparison of categorical variables was conducted using the Spearman rank test. All statistical analyses were conducted with the IBM SPSS program, version 26.0 (SPSS Inc., IBM Company, Chicago, IL, USA). For all analyses, the alpha level was set a priori to <0.05.

## 3. Results

### 3.1. Characteristics of Mothers and Infants

Table 1 presents maternal and infants’ descriptive characteristic data. In total, 136 lactating mothers were recruited with their infants. The majority (*n* = 126, 93%) were preterm infants, born at <37 weeks of gestational age (GA), and 62 infants (46%) had an extremely low birth weight (<1000 g).

### 3.2. Human Milk Composition

The HM composition is shown in Table 2.

### 3.3. Association of Maternal Nutrition with Human Milk Composition

Table 3 represents the maternal dietary nutrients and energy intake per day in correlation with the HM composition. The Spearman correlation coefficient was highly significant between the dietary protein intake (g) and true protein (*r* = 0.997; *p* < 0.001), and the protein intake (% kcal) and true protein in the HM (*r* = 0.995; *p* < 0.001). The daily dietary fat intake (when expressed in both g and % kcal) was positively correlated with the fat content in HM (*r* = 0.999; *p* < 0.001 and *r* = 0.999; *p* < 0.001, respectively). The correlation was highly significant between carbohydrate intake (g and % kcal) and carbohydrate milk content (*r* = 0.999; *p* < 0.001 and *r* = 0.998; *p* < 0.001). A significant correlation between energy intake in the diet and in the HM was detected (*r* = 1.000; *p* < 0.001).

An increased nutrient protein intake (g and % kcal) was positively correlated with the adiponectin content in HM (*r* = 0.413; *p* < 0.001 and *r* = 0.411; *p* < 0.001). Additionally, a positive correlation between dietary protein content (g and % kcal) and leptin in HM (*r* = 0.199; *p* < 0.05 and *r* = 0.191; *p* < 0.05) was found.

### 3.4. Association of Body Mass Index with Human Milk Composition

Five (3.7%) mothers were defined as underweight with a BMI < 18.5 kg/m^2^; 74 (54.4%) had a normal weight with a BMI of 18.5–24.9 kg/m^2^; 51 (37.5%) were defined as overweight with a BMI of 25.0–29.9 kg/m^2^; and six (4.4%) mothers were obese with a BMI of >30 kg/m^2^. Table 4 highlights the HM composition among the different maternal BMI categories.

The nutritional values of HM were significantly lower in underweight lactating mothers compared to normal-weight mothers in terms of true protein (*p* = 0.008), crude protein (*p* = 0.007) and adiponectin content (*p* = 0.039). Further, true protein (*p* = 0.009), crude protein (*p* = 0.008) and leptin concentrations (*p* = 0.009) were significantly lower in underweight mothers compared to overweight mothers.

The HM values in obese mothers were significantly increased in true protein (*p* = 0.042) and crude protein (*p* = 0.028), fat (*p* = 0.045) and decreased in leptin (*p* = 0.016) compared to underweight lactating mothers. Leptin (*p* = 0.002) was significantly increased in HM in overweight mothers compared to normal-weight mothers.

### 3.5. Association of Body Mass Index and Maternal Nutrition

Table 5 depicts the maternal diet in relation to maternal BMI categories. Mothers with a normal weight status had an increased protein intake compared to underweight mothers, as did normal-weight mothers compared to overweight mothers (protein in g: *p* = 0.015; protein in % of kcal: *p* = 0.009). Obese mothers showed a highly significantly increased dietary protein intake (g: *p* = 0.045; % of kcal: *p* = 0.027) and increased fat intake (g: *p* = 0.045; % of kcal: *p* = 0.045) compared to underweight mothers.

## 4. Discussion

This study demonstrates that the maternal diet has a significant impact on the HM composition. Increased dietary protein, fat, carbohydrate and energy intake of the lactating mother was associated with increased HM content with respect to these nutrients, especially with regard to protein levels, which are crucial for brain maturation and development in preterm infants. Moreover, an increased maternal BMI was positively associated with an increased protein concentration in HM. Furthermore, a higher maternal BMI was positively associated with a higher maternal dietary protein intake. Lactating mothers with a higher dietary protein intake displayed increased levels of adiponectin and leptin in their own HM. Protein is an essential macronutrient, but it also represents a challenge, especially for extremely low birthweight (ELBW) infants. The protein quantity in HM is insufficient for preterm infants; therefore, fortification is recommended. Preterm infants need approximately twice as much protein as term infants [45,46]. Studies have shown that HM feeding, particularly with a higher protein intake, is associated with better growth and neurodevelopment in preterm infants [47,48,49]. However, a high protein intake during the first few months of life is a risk factor for obesity in early childhood or adolescence [50]. It is therefore increasingly important that the individual protein needs of preterm infants are considered to achieve optimum development and prevent obesity. Hence, individualized target fortification, primarily considering the protein of HM, is fundamental. Fortifiers should only be added in specific amounts to achieve an individualized target protein content. This approach based on individualized protein target fortification on human milk analysis focusing on optimal nutrition might decrease the risk of necrotizing enterocolitis (NEC), improve growth close to intrauterine growth rates and prevent obesity risk.

Previous studies have highlighted the link between maternal nutrition considering single foods or particular nutrients and specific HM compounds but did not consider the entire maternal diet as well as the macronutrient HM content [10,17]. The finding that maternal nutrition has a strong influence on HM composition, especially on the key nutrient of protein, is particularly significant for the nutritional management of preterm infants. Mothers can actively increase the protein content in their milk by increasing their own protein intake. The increased level of protein in HM is bioavailable for preterm infants and can positively affect the growth and neurodevelopment of the infant. Further, most available human milk fortifiers contain predominantly bovine protein, which is proposed to decelerate the gastrointestinal transit time and might cause gastrointestinal discomfort [51]. Hence, preterm infants tolerate protein from HM better than that of bovine milk. Additionally, the administration of bovine protein products is proposed to increase the osmolarity of human milk, which is associated with an increased NEC incidence [52]. Additionally, fortifiers—as well as preterm infant formulas—do not contain any biologically active substances; therefore, the best choice is to use the mothers’ own milk, or donor milk provided from single preterm lactating mothers [52,53].

The milk of term lactating mothers contains the optimal protein content to ensure the adequate growth of the infant [2]. However, some mothers show delayed lactogenesis, or insufficient or even impaired lactation; other mothers decide not to breastfeed at all. Therefore, industrially manufactured infant formulas have been produced and can be used as a feeding alternative. Nevertheless, these products contain an increased protein content compared to HM and thus influence infants’ development, especially according to the early protein hypothesis. Increased protein intake during the first two years of life might be a risk factor for developing overweight and obesity in childhood and adolescence [50]. HM feeding in term infants should be encouraged considering this obesity protein leverage hypothesis.

In the present study, we report that the maternal body mass index is associated with the HM composition. We observed that lactating mothers with an increased BMI provided HM with higher levels of protein. Our findings are consistent with previous research groups providing the same positive association [9,54,55].

Fat is highly variable in HM, and we observed that obese mothers showed an increased fat concentration in HM compared to underweight mothers. These outcomes are in line with the literature stating that maternal BMI is positively associated with this macronutrient [9,18,29,56,57]. Therefore, this study suggests that maternal BMI modulates the HM composition. This should also be considered in the nutritional management of infants. However, it is not yet clear whether maternal BMI or maternal nutrition is the decisive factor or which has the most impact on the HM composition.

We detected that maternal BMI was positively associated with dietary protein intake. This highlights that increased dietary protein intake in adulthood might be also a predictor of overweight or obesity. Adiponectin and leptin are produced by adipocytes and the mammary gland and might play a key role in infants’ growth and obesity prevention. We found that adiponectin levels were higher compared to leptin levels, which was also demonstrated by another study [33]. The increased adiponectin levels in HM might decrease the risk of obesity and inflammatory disorders; therefore, breastfeeding should be promoted [58]. Other investigations evidenced that adiponectin may play a role in infants’ metabolic development, increasing insulin sensitivity and insulin action in the gut [33,59,60]. The presence of these adipose tissue markers in HM might affect infants’ short- or long-term metabolic health outcomes. However, limited information has been published and metabolic mechanisms are not completely understood, or are even considered lacking for preterm infants, illustrating the need for further studies.

Leptin and adiponectin in HM are associated with body fat and nutritional status regulation [61]. Increased levels of leptin and adiponectin are correlated with obesity [62,63,64,65,66]. In our study, overweight mothers provided HM with higher leptin levels. Savino et al. also found a positive association between maternal BMI and leptin levels in HM and hypothesized that the leptin concentration in HM might be affected by maternal mammary gland production and by the amount of leptin released from maternal body fat stores [67]. Nevertheless, the leptin amount in the maternal bloodstream could also determine the leptin concentration in HM [67]. Higher serum leptin levels correlate with a higher BMI, caused by an increased fat mass and an increased size of adipocytes. Further, leptin mRNA was detected to be more expressed in subcutaneous than in omental adipocytes [68]. The leptin levels in HM might be associated with the maternal body composition and infants’ growth and development. Moreover, they represent a regulating factor in appetite control and food intake during infants’ early lives [69]. Our study also showed that mothers with an increased protein nutrient intake had an increased leptin and adiponectin value in HM. Nevertheless, more studies on this topic are needed to evaluate the impact of maternal diet on adipose tissue markers.

This study has several strengths. One is the pooling of HM samples of individual mothers over 24 h into one representative sample that considers physiological variations in HM. In addition, HM analyses were performed by the same trained and experienced personnel. Additionally, all mothers were interviewed by the same person, avoiding response bias in the dietary evaluation. Some limitations should be considered in interpreting the results of this study. The sample size calculation was based on the primary outcome of HM processing and not on the secondary outcome parameters of the current study, which might have influenced the results. Although this study enrolled more lactating mothers compared to similar studies [9,12,16,18,66,70,71], the numbers of underweight and obese mothers were low compared to the group of normal-weight and overweight mothers. Thus, it is important that future studies thoroughly compare the different maternal BMI categories to provide a better insight, especially regarding how underweight and obese lactating mothers differ in their HM compositions compared to normal-weight mothers. The inclusion criteria were met if the mother breastfed or pumped for her infant, regardless of whether the infant was premature or term. More preterm lactating mothers took part in this study, which could be because these mothers were more interested in knowing the composition of their HM than mothers who gave birth to healthy term infants. In addition, the coronavirus pandemic might be another driving factor; mothers of healthy full-term infants were not motivated to take part in this trial and to return to the hospital for HM delivery and the 24-h recall after discharge.

The lactation period affects the HM nutrient composition, especially protein [12]. In our study sample, a large range of lactation periods was observed. However, overweight or obese mothers often have problems with lactation, and many preterm mothers suffer from delayed lactogenesis [72,73]. Therefore, lactating mothers with sufficient milk production were invited to take part in this study.

Adiponectin and leptin measurements could not be carried out in all samples because there were insufficient volumes in fifteen HM samples. As with other studies on this topic, we used the standard BMI categories for adults. Specific cut-off levels for post-delivery BMI classification for women are not available. However, it must be mentioned that lactating mothers show an increased BMI due to the recent pregnancy, and recommended weight gain ranges for pregnant women are based on maternal pre-pregnancy BMI [74]. Therefore, future studies should consider the pre-pregnancy BMI, the actual weight gain during pregnancy and a comparison to the recommended weight gain range in kg [74] to acquire an overview of the nutritional state during the entire pregnancy.

## 5. Conclusions

Adequate and personalized maternal nutrition during lactation is crucial to provide the newborn infant with the mother’s own milk containing the optimal amount of nutrients. A personalized diet with a high protein intake may play an important role in the long-term health of infants of lactating mothers. Furthermore, the body mass index of lactating women might also play a role in infant obesity prevention. It is therefore even more important that the individual protein needs of infants are considered to achieve optimum development and to prevent obesity. Hence, individualized target fortification, primarily considering the protein of HM, is fundamental.

The knowledge of the maternal diet and maternal BMI impacting the HM composition is essential to optimize the feeding of newborn infants. This is especially relevant in the nutritional management of preterm infants and can be utilized for approaches to improve growth rates and appropriate development based on individualized protein target fortification. HM feeding should be encouraged considering the obesity protein leverage hypothesis.

## Figures and Tables

**Table 1:** Mothers’ and infants’ characteristics.

Characteristic	*n* = 136
***Maternal characteristics***	
Age of mother (years)	33 (27–36)
Height (cm)	167 (162–172)
Weight (kg)	68 (±10)
BMI (kg/m^2^)	24.3 (22.0–27.1)
Lactation (days)	29 (14–66)
Breastfeeding/pumping per day	6 (4–7)
Primiparous, *n* (%)	77 (56.6)
Cesarean delivery, *n* (%)	124 (91.2)
***Infants’ characteristics***	
Male sex, *n* (%)	76 (55.9)
Preterm infants < 37 weeks of GA, *n* (%)	126 (92.7)
Gestational age (wk + d)	29 + 3 (26 + 2–33 + 5)
Birth weight (g)	1063 (845–2269)
Birth height (cm)	35.5 (33.5–45.0)
Head circumference (cm)	26.0 (24.0–31.9)

Results are presented as mean (±SD), median (IQR) or as number (%). SD: standard deviation; IQR: interquartile range. BMI: body mass index; GA: gestational age; wk: week; d: day.

**Table 2:** Human milk nutrient composition.

Parameter	Human Milk (*n* = 136)
True protein (g/100 mL)	1.10 (0.90–1.40)
Crude protein (g/100 mL)	1.30 (1.06–1.65)
Fat (g/100 mL)	2.96 (±0.87)
Carbohydrate (g/100 mL)	7.70 (7.00–8.00)
Energy (kcal/100 mL)	63.19 (±9.80)
Total solids (g/100 mL)	12.15 (±1.31)
Adiponectin (ng/mL)	18.90 (13.60–25.45)
Leptin (ng/mL)	0.108 (0.008–0.314)

Results are presented as mean (±SD) or median (IQR). SD: standard deviation; IQR: interquartile range. Adinopectin and leptin (*n* = 121); all other parameters (*n* = 136).

**Table 3:** Correlation between maternal nutrition and human milk composition.

Mothers’ Nutrient and Energy Intake/d		Human Milk Composition															
		True Protein (g/100 mL)		Crude Protein (g/100 mL)		Fat (g/100 mL)		Carbohydrate (g/100 mL)		Energy (kcal/100 mL)		Total Solids (g/100 mL)		Adiponectin (ng/mL)		Leptin (ng/mL)	
Parameter	Median (IQR)	*r*	*p*	*r*	*p*	*r*	*p*	*r*	*p*	*r*	*p*	*r*	*p*	*r*	*p*	*r*	*p*
Protein (g)	59.9 (44.1–100.2)	0.997	**<0.001**	0.995	**<0.001**	0.184	**0.032**	0.369	**<0.001**	0.463	**<0.001**	0.531	**<0.001**	0.413	**<0.001**	0.199	**0.035**
Protein (% kcal)	16.0 (14.0–19.0)	0.995	**<0.001**	0.996	**<0.001**	0.186	**0.031**	0.361	**<0.001**	0.462	**<0.001**	0.530	**<0.001**	0.411	**<0.001**	0.191	**0.040**
Fat (g)	96.4 (81.4–118.3)	0.205	**0.017**	0.208	**0.015**	0.999	**<0.001**	0.029	0.740	0.878	**<0.001**	0.776	**<0.001**	0.028	0.758	0.109	0.235
Fat (% kcal)	43.0 (37.3–48.0)	0.206	**0.016**	0.209	**0.015**	0.999	**<0.001**	0.034	0.698	0.879	**<0.001**	0.777	**<0.001**	0.031	0.733	0.108	0.238
Carbohydrates (g)	197.1 (151.8–240.6)	0.375	**<0.001**	0.373	**<0.001**	0.050	0.560	0.999	**<0.001**	0.352	**<0.001**	0.556	**<0.001**	0.165	0.070	−0.039	0.670
Carbohydrates (% kcal)	40.0 (34.3–47.0)	0.375	**<0.001**	0.374	**<0.001**	0.054	0.536	0.998	**<0.001**	0.354	**<0.001**	0.558	**<0.001**	0.164	0.073	−0.037	0.689
Energy (kcal)	1966.1 (1781.9–2320.3)	0.466	**<0.001**	0.468	**<0.001**	0.866	**<0.001**	0.338	**<0.001**	1.000	**<0.001**	0.944	**<0.001**	0.092	0.315	0.159	0.081

Data on mothers’ diet are presented as median (IQR). IQR: interquartile range. *r* = correlation coefficient, *p* = level of significance. The correlation between diet and milk composition was determined using Spearman’s correlation analysis. Results of human milk composition are presented as Spearman’s *r* coefficients. Bold values indicate that correlation is significant at the 0.05 level (2-tailed). Adinopectin and leptin (*n* = 121); all other human milk parameters (*n* = 136).

**Table 4:** Human milk nutrient composition compared in different maternal BMI categories.

Parameter	BMI < 18.5 kg/m^2^ (*n* = 5)	BMI 18.5–24.9 kg/m^2^ (*n* = 74)	BMI 25–29.9 kg/m^2^ (*n* = 51)	BMI > 30 kg/m^2^ (*n* = 6)
True protein (g/100 mL)	0.75 (±0.15) ^a,b^	1.10 (0.90–1.41)	1.05 (0.90–1.40)	1.06 (±0.25) ^c^
Crude protein (g/100 mL)	0.90 (±0.19) ^a,b^	1.30 (1.10–1.73)	1.30 (1.10–1.70)	1.31 (±0.27) ^c^
Fat (g/100 mL)	2.50 (±0.80)	2.97 (±0.90)	2.93 (±0.81)	3.52 (±1.03) ^c^
Carbohydrate (g/100 mL)	7.51 (±0.23)	7.80 (6.84–8.00)	7.70 (7.15–8.00)	7.57 (±0.41)
Energy (kcal/100 mL)	57.10 (±8.26)	62.96 (±10.23)	63.54 (±9.00)	68.25 (±11.55)
Total solids (g/100 mL)	11.22 (±0.94)	12.11 (±1.35)	12.25 (±1.24)	12.68 (±1.37)
Adiponectin (ng/mL)	14.22 (±3.37) ^a^	21.75 (14.98–26.40)	18.80 (11.5–23.9)	16.46 (±7.55)
Leptin (ng/mL)	0.019 (±0.03) ^b^	0.063 (0.000–0.203)	0.195 (0.045–0.391) ^d^	0.182 (±0.111) ^c^

Results are presented as mean (±SD) or median (IQR). SD: standard deviation; IQR: interquartile range. BMI: body mass index. Adinopectin and leptin (*n* = 121); all other parameters (*n* = 136). ^a^ BMI < 18.5 kg/m^2^ versus BMI 18.5–24.9 kg/m^2^ group, *p* < 0.05. ^b^ BMI < 18.5 kg/m^2^ versus BMI 25.0–29.9 kg/m^2^ group, *p* < 0.01. ^c^ BMI > 30 kg/m^2^ versus BMI <18.5 kg/m^2^ group, *p* < 0.05. ^d^ BMI 25.0–29.9 kg/m^2^ versus BMI 18.5–24.9 kg/m^2^ group, *p* < 0.01.

**Table 5:** Maternal nutritional intake related to different maternal BMI categories.

Mothers’ Nutrient and Energy Intake/d	BMI < 18.5 kg/m^2^ (*n* = 5)	BMI 18.5–24.9 kg/m^2^ (*n* = 74)	BMI 25–29.9 kg/m^2^ (*n* = 51)	BMI > 30 kg/m^2^ (*n* = 6)
Protein (g)	41.3 (±3.2)	62.4 (44.2–101.0) ^a,b^	56.3 (44.0–100.4)	67.3 (±21.0) ^c^
Protein (% kcal)	12.0 (±3.0)	17.0 (±5.0)	16.0 (14.0–19.0)	17.0 (14.0–18.0) ^c^
Fat (g)	73.5 (67.2–107.8)	98.2 (81.9–118.3)	96.0 (79.1–115.9)	122.6 (±41.7) ^c^
Fat (% kcal)	38.0 (±8.0)	45.0 (38.0–48.0)	43.0 (37.0–48.0)	50.0 (±14.0)
Carbohydrates (g)	186.0 (182.2–209.5)	198.3 (±70.6)	201.3 (169.4–241.3)	202.4 (±38.3)
Carbohydrates (% kcal)	38.0 (37.5–42.5)	39.0 (±11.0)	40.0 (37.0–48.0)	41.0 (±5.0)
Energy (kcal)	1841.0 (±306.1)	2064.6 (±481.2)	1950.8 (1785.5–2277.5)	2391.6 (±635.2)

Results are presented as mean (±SD) or median (IQR). SD: standard deviation; IQR: interquartile range. BMI: body mass index. ^a^ BMI 18.5–24.9 kg/m^2^ versus BMI < 18.5 kg/m^2^ group, *p* < 0.05. ^b^ BMI 18.5–24.9 kg/m^2^ versus BMI 25.0–29.9 kg/m^2^ group, *p* < 0.01. ^c^ BMI > 30 kg/m^2^ versus BMI < 18.5 kg/m^2^ group, *p* < 0.05.

## Data Availability

The data that support the findings of this trial are available upon reasonable request.
